# The effect of induced mutations on quantitative traits in *Arabidopsis thaliana*: Natural versus artificial conditions

**DOI:** 10.1002/ece3.2558

**Published:** 2016-10-24

**Authors:** Frank W. Stearns, Charles B. Fenster

**Affiliations:** ^1^ Department of BiologyBiology‐Psychology BuildingUniversity of MarylandCollege ParkMDUSA

**Keywords:** *Arabidopsis*, EMS, fitness, mutations, quantitative traits

## Abstract

Mutations are the ultimate source of all genetic variations. New mutations are expected to affect quantitative traits differently depending on the extent to which traits contribute to fitness and the environment in which they are tested. The dogma is that the preponderance of mutations affecting fitness will be skewed toward deleterious while their effects on nonfitness traits will be bidirectionally distributed. There are mixed views on the role of stress in modulating these effects. We quantify mutation effects by inducing mutations in *Arabidopsis thaliana* (Columbia accession) using the chemical ethylmethane sulfonate. We measured the effects of new mutations relative to a premutation founder for fitness components under both natural (field) and artificial (growth room) conditions. Additionally, we measured three other quantitative traits, not expected to contribute directly to fitness, under artificial conditions. We found that induced mutations were equally as likely to increase as decrease a trait when that trait was not closely related to fitness (traits that were neither survivorship nor reproduction). We also found that new mutations were more likely to decrease fitness or fitness‐related traits under more stressful field conditions than under relatively benign artificial conditions. In the benign condition, the effect of new mutations on fitness components was similar to traits not as closely related to fitness. These results highlight the importance of measuring the effects of new mutations on fitness and other traits under a range of conditions.

## 1 Introduction

Mutations are the ultimate source of all genetic variation (Nei, 2013). Because of this, the study of adaptation genetics historically modeled the process of adaptation as occurring due to novel beneficial mutations (Fisher, 1930). However, adaptation can also occur by selection acting on standing genetic variation from accumulated mutations (Barrett & Schluter, 2008; Karasov, Messer, & Petrov, 2010). The question of great importance is whether adaptation is mutation limited or selection limited. At heart is the belief that the waiting time for new beneficial mutations is too long and their effects too small for mutations to adequately contribute to adaptation to environmental changes under relatively short ecological time scales. Additionally, new mutations of traits that are closely associated with fitness are typically believed to be deleterious far more often than beneficial (Camara, Ancell, & Pigliucci, 2000; Keightley & Lynch, 2003).

Two major experimental approaches have been used to study the effects of mutations on fitness (van Harten, 1998; Kondrashov & Kondrashov, 2010). First, mutation accumulation (MA) studies reduce the strength of selection on experimental populations, allowing spontaneous mutations to accumulate by drift (Halligan & Keightley, 2009; Mukai, 1964). As selection is reduced, all but the most strongly deleterious mutations will be represented. This approach has the advantage of investigating spontaneous mutations, representative of the types of mutations that are produced under natural conditions, thus circumventing biases in the types of mutations, which are typically induced through mutagenesis. The second approach is chemical or radiation mutagenesis (Auerbach, 1949; Jambhulkar, 2007; Singer & Kusmierek, 1982). Although this approach does bias the spectrum of mutation types dependent on the mutagenizing agent, mutagenesis has the advantage of being much quicker than MA approaches based on spontaneous naturally occurring mutations. The spontaneous mutation rate is roughly one to four mutations per zygote in many of the model organisms studied (e.g., Ossowski et al., 2010). Because many more mutations can be induced in a single bout of mutagenesis, the cumulative effect of many mutations can be more readily quantified.

Recent work with *Arabidopsis thaliana* (MacKenzie, Saade, Le, Bureau, & Schoen, 2005; Rutter, Shaw, & Fenster, 2010; Shaw, Byers, & Darmo, 2000) and *Saccharomyces cerevisiae* (Hall & Joseph, 2010) has indicated that mutations may be beneficial more often in these two organisms than has been quantified in previous studies of the distribution of mutation effects on fitness (Keightley & Lynch, 2003). In particular, mutant lines in *A. thaliana* were found to increase fitness components relative to a premutated founder nearly half the time under both greenhouse (MacKenzie et al., 2005; Shaw et al., 2000) and field conditions (Rutter et al., 2010). This suggests that new mutations may be able to contribute to adaptation more quickly than previously assumed, although more study is needed before we can unequivocally state that beneficial mutations occur at high frequency.

The effects of new mutations on fitness are expected to differ more in the wild than in artificial environments. Under natural conditions, many more mutations will affect fitness than in more controlled environments (Rutter et al., 2010). In field experiments, the expectation is that more mutations will be involved in fitness, and they will have a greater effect on fitness. Stressful conditions are also known to increase the variance in mutations’ effects on fitness (Martin & Lenormand, 2006) and to result in mutations being more deleterious on average (Kondrashov & Houle, 1994 but see Chang & Shaw, 2003). For example, inbreeding depression is generally greater in more stressful versus less stressful environments (Dudash, 1990; Frankham, 2015; but see Waller, Dole, & Bersch, 2008; Agrawal & Whitlock, 2010). Furthermore, interactions among the experimental mutations and between the mutations and the environment may be complex and it is possible that higher environmental variance may mask the effects of individual mutations (Jaenike, 1982).

Here, we use chemical mutagenesis to generate mutant lines of the model plant organism *A. thaliana* (Camara & Pigliucci, 1999; Camara et al., 2000; Salinas & Sanchez‐Serrano, 2006) and plant them under field conditions alongside the premutation founder. We also planted the same lines and founder under artificial (growth room) conditions. In this way, we were able to gauge the magnitude of fitness changes through mutation in the field and the growth room, two environments with contrasting survivorship (growth room ≫ field), suggesting different degrees of stress. We also measured traits less closely related to fitness in the growth room experiment to compare the distribution of mutation effects for fitness components versus quantitative traits that are not as closely related to fitness in artificial conditions. There is an expectation that new mutations will affect such traits differently, increasing variance in either direction as opposed to being skewed toward a decrease in fitness (Keightley & Lynch, 2003).

Here, we address three questions: (1) What is the distribution of fitness effects of mutations derived from mutagenesis? (2) Does the distribution of fitness effects differ between field and relatively benign laboratory environment conditions? (3) Does the distribution of mutation effects for fitness proxies differ from the distribution for traits that are less likely to be related to fitness? To date, three laboratories have used *A. thaliana* lines and have demonstrated a much higher frequency of beneficial mutations (MacKenzie et al., 2005; Rutter et al., 2010; Shaw et al., 2000) than expected (Keightley & Eyre‐Walker, 2010; Keightley & Lynch, 2003). However, because of the much longer timespan between generations, *A. thaliana* mutation accumulation experiments represent one‐half order to an order of magnitude fewer generations than mutation accumulation experiments conducted with shorter lived organisms (Hall & Joseph, 2010; Halligan & Keightley, 2009; Katju, Packard, Bu, Keightley, & Bergthorsson, 2015; Keightley et al., 2009; Latta et al., 2015). Thus, using mutagenic approaches we hoped to not only add another field‐based estimate of mutation effects on fitness, but also to ask what the cumulative fitness effects of mutations might be when many more are generated, corresponding to other mutation accumulation experiments with shorter generation times.

## 2 Methods

### 2.1 Mutagenesis

Mutations were induced in the Columbia accession of *A. thaliana* using ethylmethane sulfonate (EMS). EMS is an alkylating agent that most commonly results in G:C to A:T substitutions (transitions) (Greene et al., 2003). Overall, EMS induces a spectrum of point mutations similar to spontaneous mutations, although it does not produce indels (Greene et al., 2003). Mutation rate was evaluated by exposing the lines to 0, 20, 30, 40, and 50 μmol/L solutions and estimating the percent of siliques with albino seeds from a sample of each treatment (17.65%, 35.71%, 34.04%, 67.50%, and 81.25%, respectively), a common measure of mutation rate in *A. thaliana* (Camara et al., 2000). The 20 μmol/L dosage was chosen in order to generate enough mutations for a measurable effect on fitness.

We estimate 25 mutations per cell in coding regions per genome, which are expected to affect a wide range of quantitative traits (Brock, 1976; Camara et al., 2000). This estimate is based on the measured rate of mutations induced by EMS, which is approximately 3.7 × 10^−6^ locus^−1^ cell^−1^ μM^−1^ hr^−1^ (Camara et al., 2000; Korneef, Dellaert, & van der Veen, 1982). *Arabidopsis thaliana* has about 28,000 loci (Redei & Koncz, 1992). This mutation rate resulted in an approximate threefold to fourfold greater number of nonsynonymous mutations in protein coding sequence as quantified from direct sequencing (Ossowski et al., 2010) of Columbia mutation accumulation lines representing 30 generations of spontaneous mutation accumulation (Ossowski et al., 2010). These sequenced lines represented a subset of the mutation accumulation lines tested for field performance (Rutter et al., 2010, 2012). Using a 20 μmol/L dosage was considered ideal because it generated visible mutations, but not so many mutations that their cumulative effects would be devastating and result in low seedling and later life‐history stage survivorship. Furthermore, the dosage resulted in meeting our criteria of generating many more mutations than were quantified in previous *A. thaliana* mutation accumulation line experiments conducted in the field, roughly equivalent to 90–120 generations of MA.

**Figure 1:**
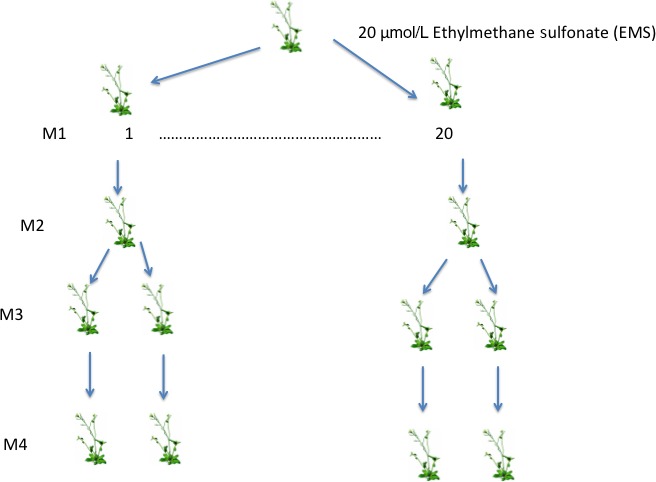
A schematic representation showing the process of generating mutation sublines from *Arabidopsis thaliana*. Seeds were collected from a single Columbia founder and treated with 20 μmol/L ethylmethane sulfonate for 12 hr. Twenty mutant lines were derived from this treatment. The lines were then split into two sublines each (M3 generation), and seeds from these sublines (via selfing) were planted in the field (Beltsville Experimental Agricultural Station (UMD) in Beltsville MD (N 39.05378 W −76.95387) or in the growth room at the University of Maryland College Park campus

After the 20  μmol/L solution of EMS was determined as the optimal concentration, seeds were washed with a 0.1% solution of Tween‐20 and then soaked for 12 hr in EMS with rotation. The seeds were next washed and soaked in distilled water for 6 hr with rotation. After washing, the seeds were sown directly onto soil in individual pots (Figure 1). Seeds from the premutated founder were simultaneously sown so as to generate seeds to represent the founder generation. These mutagenized seeds and seeds sown from the premutated founder were cold treated at 2°C for two weeks with no light. They were then allowed to germinate on benches in a growth room at 20°C with 24 hr of fluorescent light and 8 hr of incandescent light for 2 weeks. After this time, individual seedlings were transplanted to their own pots to generate the founder and the 20 mutant lines. This was the M1 generation. These plants were grown in the growth room under the same conditions used for germination (20°C with 24 hr of fluorescent light and 8 hr of incandescent light). Seed was collected from these plants and sown out as before, with 2 weeks at 2°C with no light and then moved to a growth room to germinate at 20°C with 24 hr of fluorescent light and 8 hr of incandescent light for 2 weeks as before. Individual seedlings were isolated into pots, 20 individuals per mutation line and premutant founder. This was the M2 generation. These plants were again allowed to grow in the growth room under the same conditions used for germination (20°C with 24 hr of fluorescent light and 8 hr of incandescent light). Seed was collected from these plants. This seed was treated as before to induce germination. At transplant, each of the 20 replicates from the 20 mutant lines and the founder were split into two sublines by transplanting seedlings into individual pots. The sublines were grown in random locations in the growth room to minimize biases due to maternal effects introduced by the specific location within the greenhouse. This was the M3 generation. These plants were again grown in the growth room at 20°C with 24 hr of fluorescent light and 8 hr of incandescent light. Seed collected from these individuals was the M4 generation. Individuals from this generation along with founder lines were used for fitness assessment in the field.

### 2.2 Fitness assessment—field conditions

Premutated and mutated seeds from among the founder and mutant lines, respectively, were planted during Fall of 2013 at the Beltsville Experimental Agricultural Station (UMD) in Beltsville MD (N 39.05378 W −76.95387). Weather data records for the Beltsville Experimental Agriculture Station can be found here: http://www.ba.ars.usda.gov/weather/ba-weather-2.html. Prior to planting, seeds were cold treated for 2 weeks at 2°C and allowed to germinate on benches at 20°C with 24 hr of fluorescent light and 8 hr of incandescent light for 2 weeks, as above. Seedlings were then transplanted in random order into plug trays and allowed two more weeks to establish; 20°C with 24 hr of fluorescent light on a bench and 8 hr of incandescent light for 1 week and then at 2°C with 8 hr of incandescent light for another week in order to cold acclimate them. Plug trays were transported to the field, and then, the seedlings were transplanted into the field with their soil plugs. Seedlings were spaced 10 cm apart, maintaining the spatial orientation of seedlings in the plug trays. In this experiment, 60 seedlings from each of the two sublines for each of the 20 mutant lines and the founder were planted, for a total of 2,520 plants. When planted, the seedlings were at the 2‐ to 4‐leaf stage. The plots were initially watered at planting to facilitate establishment but otherwise were exposed to natural weather conditions, pathogens, predation from herbivores, competition with other plant species, and other natural exigencies. All experimental plants were harvested above ground at the end of May 2014, when plants were in the senescent phase. Harvested plants were dried in heat chambers. Aboveground dry mass and total fruit number were measured from a sample for each experiment, and aboveground dry mass was found to be a good predictor of fruit number (*r* ^2^ = .89, *n* = 15). We therefore used dry mass of survivors multiplied by proportion of plants surviving to harvest as our measure of fitness for each mutant line and premutant founder. This is a reasonable proxy of fitness for a selfing annual plant (Shaw et al., 2000).

### 2.3 Growth room

Plants from the same lines were grown in a walk‐in growth room at the University of Maryland. The same experimental design was sowed into plug trays in Spring 2014. Seeds were cold treated for 2 weeks at 2°C and allowed to germinate on benches at 20°C with 24 hr of fluorescent light and 8 hr of incandescent light for 2 weeks, as above. These plants were cultivated in the growth room at 20°C with 24 hr of fluorescent light. Seedlings were then transplanted in random order into plug trays (give size of the plug) and continued to grow at 20°C with 24 hr of fluorescent light on a bench and 8 hr of incandescent light. A total of 15–25 plants from each of the two sublines and the founder sublines were grown in three blocks and were measured for four traits: silique number (=number of fruit), Julian day of first flower, number of trichomes per mm of midrib length, and the ratio of side branch mass to main branch mass. They were allowed to grow from April 2014 to December 2014. All plants were harvested above ground when they went to seed unless they had not bolted by December 2014, at which point all remaining plants were harvested. Because we were not certain whether these plants would have bolted or not, they were left out of measurements that relied on bolting (silique number, Julian day of first flower, and the ratio of side branch weight to mid‐branch weight). These plants represented about 6% of the overall plants measured. Julian day of first flower was assessed daily. The largest leaf at first flower was collected and used to determine trichome number per midrib length. Aboveground biomass was collected at the time plants went to seed. Silique number and the mass of the main branch and side branches were measured on the dried samples. We consider silique number to be a fitness proxy.

### 2.4 Statistical analysis

#### 2.4.1 Field

Statistical analyses were conducted in R (R Core Team, 2014) using the package STATS. Fitness (survivorship × plant mass) was examined for significant mutant line effects. Because fitness of a line included many replicates that did not survive, the data consisted of many zeros; consequently, the nonparametric Kruskal–Wallis test was used, a conservative assay for testing line effects. We used the model Fitness = Line + Error. Again, because of low field survivorship, we used a nonparametric test to determine whether the founder phenotype or performance differed significantly from the mean of the mutant lines. We used a sign test (Whitlock & Schluter, 2009) to determine for each measured trait if the rank of the founder as compared to the mutant lines differed from the null expectation of no difference, that is, that the rank order of the founder is equal to the median of the mutant lines.

#### 2.4.2 Growth room

Statistical analyses were conducted in R (R Core Team, 2014) using the package STATS except for the negative binomial GLM which used the package MASS (see Appendix A for R codes). All traits (Julian day of flowering, silique number, trichome number per midrib length, and proportion of side branch/main branch mass were examined for significant mutant line effects. Different analyses were used depending on the character. Flowering time was normally distributed and a one‐way ANOVA was performed. Silique number was examined with a negative binomial general linearized model (GLM). Trichome number per midrib length was gamma distributed but contained many zeroes, so was also analyzed with a Kruskal–Wallis test. Side branch/main branch mass is a continuous trait with a high number of zeros, so the nonparametric Kruskal–Wallis test was used here as well. All tests used the model Trait = Line + Error. To determine whether the founder phenotype or performance differed significantly from the mean of the mutant lines, sign tests were used for all traits to determine whether the rank of the founder differed from the null expectation of no difference, that is., the rank order of the founder = the median of the mutant lines. To determine whether the cumulative effects of mutations were correlated among the measured traits, that is, pleiotropic, we quantified Pearson's product–moment correlations among those traits demonstrating significant line effects, using each mutant line as a replicate. As multiple tests were conducted with the same data set derived from the growth room experiments, all results from the growth room were sequentially Bonferroni corrected (Whitlock & Schluter, 2009) to provide an appropriate Type I error rate.

Because of overall low survivorship in the field and limited replication for the growth room experiments, we collapsed subline effects to line effects; that is, we pooled our sublines. The generation of the sublines occurred in random locations in the growth room, and so by pooling sublines, we control for the effect of specific location of maternal plant on seed quality. By not utilizing sublines in our analyses of the performance of seed from these sublines, we likely inflate the environmental variance, making any finding of mutant line effects more robust. In other words, by pooling sublines we control for microenvironmental or maternal effects on seed quality but do not remove these effects from the effect of line and therefore likely increase the error component contributing to performance or trait expression.

## 3 Results

### 3.1 Field

**Figure 2:**
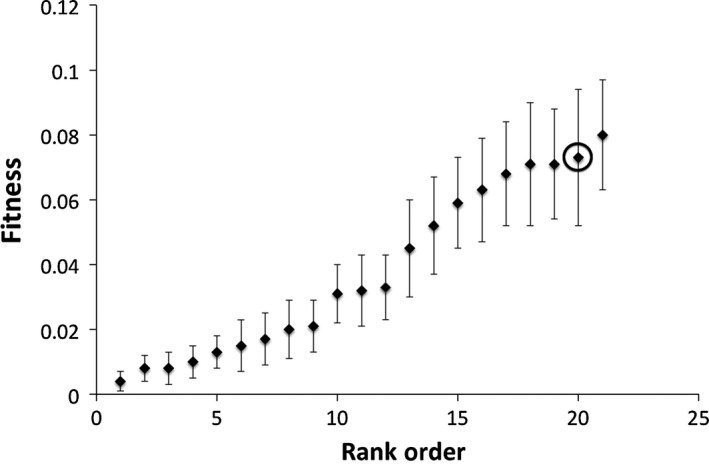
The mean fitness (mass x survivorship) for *Arabidopsis thaliana* under field conditions at the Beltsville Experimental Agricultural Station (UMD) in Beltsville MD (N 39.05378 W −76.95387) listed in rank order for the premutation founder and 20 mutant lines generated from ethylmethane sulfonate (20 μmol/L) mutagenesis (planted: *n* = 120; weighed: mutant line average sample size *n* = 12.5, σ of sample size = 6.57, founder *n* = 17). A mutant line effect was detected (*p* < .001). The premutation founder is circled. All but one mutant line showed reduced fitness compared to the founder (*p* < .001). Error bars indicate one standard error from the mean

Under field conditions, there was a significant mutation line effect on fitness (Kruskal–Wallis *Χ* ^2^ = 85.11, *df* = 19, *p* < .001), indicating that induced mutations contributed to among line variance. The Columbia founder line was found to have the second highest fitness (and the highest mass when survivorship was not included in the data) (Figure 2). A sign test of the performance of the mutant lines relative to the founder indicated that the founder had significantly higher fitness than the median of the mutant lines (*p* < .001, *n* = 21).

### 3.2 Growth room

**Figure 3:**
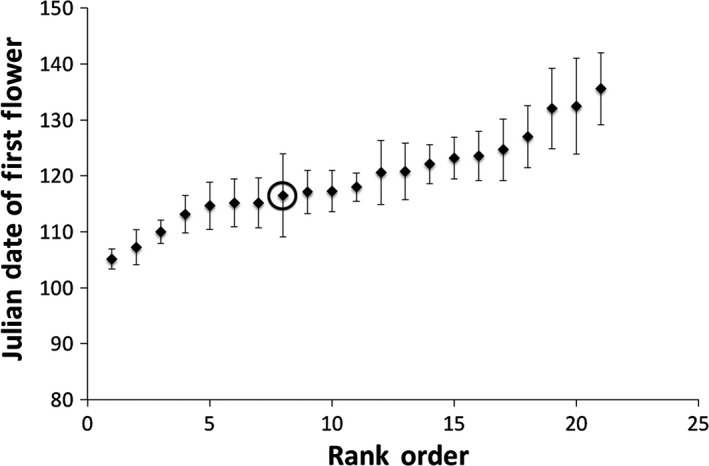
The mean Julian day of first flower for *Arabidopsis thaliana* under growth room conditions (20°C, 8 hr of incandescent light) on the University of Maryland College Park campus. Means are presented in rank order for the premutation founder and 20 mutant lines generated from ethylmethane sulfonate (20 μmol/L) mutagenesis (mutant line mean sample size *n* = 15.21, σ of sample size = 3.84; founder *n* = 6). A mutant line effect was detected (*p* < .001). The premutation founder is circled. Error bars indicate one standard error from the mean

**Figure 4:**
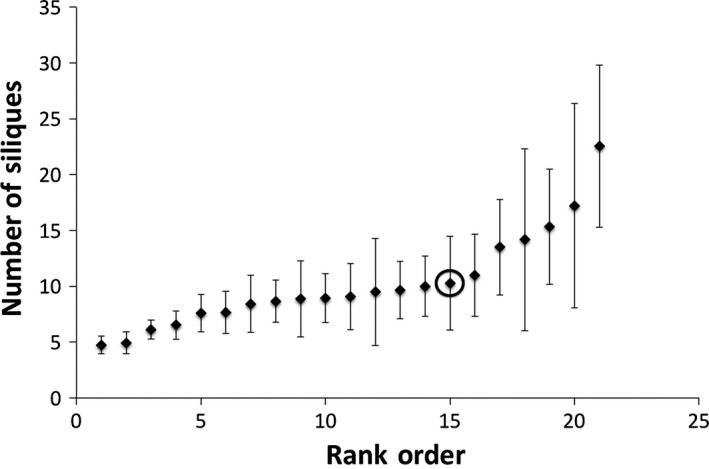
The mean number of siliques for *Arabidopsis thaliana* under growth room conditions (20°C, 8 hr of incandescent light) on the University of Maryland College Park campus. Means are presented in rank order for the premutation founder and 20 mutant lines generated from ethylmethane sulfonate (20 μmol/L) mutagenesis (mutant line mean sample size *n* = 14.80, σ of sample size = 4.12; founder *n* = 7). A mutant line effect was detected (*p* < .001). The premutation founder is circled. Error bars indicate one standard error from the mean

**Figure 5:**
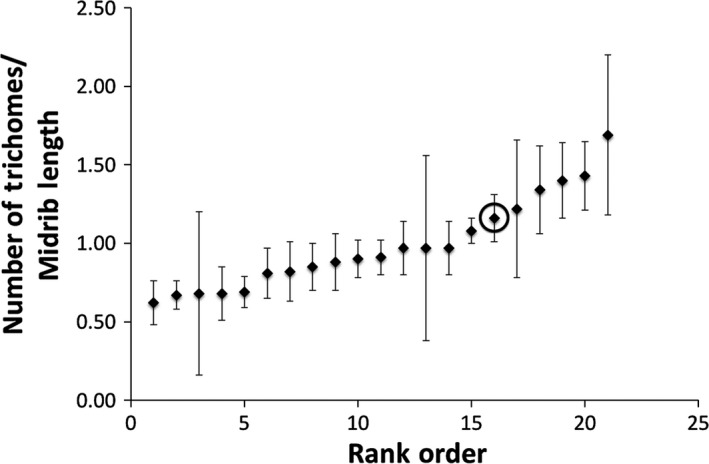
The mean number of trichomes per midrib length (mm) for *Arabidopsis thaliana* under growth room conditions (20°C, 8 hr of incandescent light) on the University of Maryland College Park campus. Means, in rank order, are presented for the premutation founder and 20 mutant lines generated from ethylmethane sulfonate (20 μmol/L) mutagenesis (mutant line mean sample size *n* = 10.65, σ of sample size = 3.73; founder *n* = 7). The effect of mutant line on the trait was not significant after sequential Bonferroni correction (*p* = .025, α = 0.0125). The premutation founder is circled. Error bars indicate one standard error from the mean

**Figure 6:**
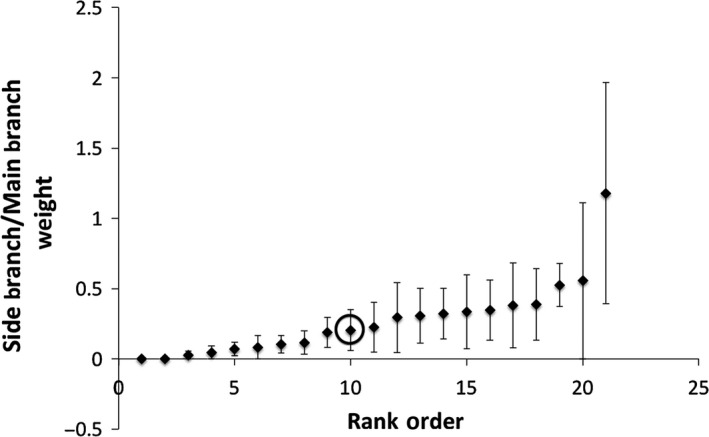
The mean proportion of side branches/main branch mass for *Arabidopsis thaliana* under growth room conditions (20°C, 8 hr of incandescent light) on the University of Maryland College Park campus. Means are presented in rank order for the premutation founder and 20 mutant lines generated from ethylmethane sulfonate (20 μmol/L) mutagenesis (mutant line mean sample size *n* = 12.40, σ of sample size = 2.89; founder *n* = 7). Plants without side branches were given a side branch mass of 0. There was no significant effect of mutant line on the trait (*p* = .31). The premutation founder is circled. Error bars indicate one standard error from the mean

There was a significant mutant line effect for day of first flowering (*df* = 19, *p *< .001) (Figure 3), siliques (*df* = 19, *p* < .001) (Figure 4), and trichome number (Kruskal–Wallis *Χ* ^2^ = 32.09, *df* = 19, *p* = .031) (Figure 5), indicating that among line variance in these traits could be attributed to induced mutations. There was no significant line effect for side branch/main branch mass (Kruskal–Wallis *Χ* ^2^ = 22.45, *df* = 19, *p* = .26) (Figure 6). After sequential Bonferroni correction, there was an effect for day of first flower and for silique number, but trichome number only trended toward significant (*p* = .06). A sign test only indicated a significant deviation from the median value of the mutant lines for the founder for number of trichomes per midrib length (*p* = .04, *n* = 21), the founder had more trichomes than 15 of the 20 mutant lines, but this was not significant after sequential Bonferroni correction. The rank of all other traits was not significant. In other words, there is little evidence of the mean character state of the mutant lines differing from the founder for the four traits measured in the growth room.

There were no significant correlations between pairs of traits that demonstrated significant line effects (all values *p* > .29, *n* = 21).

## 4 Discussion

Mutations are expected to decrease fitness more often, but to have bidirectional or symmetrical effects on traits that are not closely related to fitness (Camara & Pigliucci, 1999; Keightley, Davies, Peters, & Shaw, 2000). Using mutant lines of the Columbia strain of *A. thaliana* derived from EMS mutagenesis and measuring four traits in a growth room, as well as fitness characters in the field, we found that mutations were more likely to decrease fitness in the field, than unrelated characters under artificial growth conditions. Significant mutant line effects suggest that measured differences in these traits were due to induced mutations. These results are consistent with the notion that most mutations are deleterious (Keightley & Lynch, 2003) but conflict with recent results in studies of *A. thaliana* mutant lines (MacKenzie et al., 2005; Rutter et al., 2010; Shaw et al., 2000).

### 4.1 Fitness

Earlier work on *A. thaliana* mutation accumulation lines, as well as on mutation accumulation lines from other species, has demonstrated that mutations can be beneficial more often than previously believed (Hall & Joseph, 2010; MacKenzie et al., 2005; Rutter et al., 2010; Shaw et al., 2000). In *A. thaliana*, mutation accumulation lines have been shown to have increased fitness over the premutation founder as much as 1/3 to 1/2 the time (MacKenzie et al., 2005; Rutter et al., 2010; Shaw et al., 2000), corresponding to high beneficial mutation rates. However, here we found that most mutant lines (19/20) had reduced fitness relative to the premutation founder under field conditions. In the more benign conditions of the growth room, the difference between founder and mutant lines was much less pronounced. Previous work on an earlier generation of these mutant lines derived from EMS mutagenesis found a similar result: In two of three field plantings, mutations reduced fitness on average relative to the founder (Stearns & Fenster, 2016).

The difference between the mutation accumulation studies and our chemical mutagenesis study may be explained in two ways. First, the estimate of the number of mutations induced in coding regions, and hence potentially affecting fitness, was far greater than the number of mutations accumulated in the studies of spontaneous mutations (25 here via mutagenesis vs. four per line via spontaneous mutations in Ossowski et al., 2010 as determined by direct sequence). The increase in the number of mutations potentially affecting fitness increases the probability that a large magnitude deleterious mutation will affect the line (Camara et al., 2000) and lead to a genetic death (Crow, 1997, 2000; Muller, 1950); that is, if mutations that have a strong deleterious effect on fitness occur with some regularity, increasing the number of mutations increases the chance that a line will get one of these mutations, and it will swamp the effects of any slightly beneficial mutations, dragging the fitness of the line down. This has been corroborated experimentally (Davies, Peters, & Keightley, 1999) and most recently by Heilbron, Toll‐Reira, Kojadinovic, and MacLean (2014) who found that 42.3% of the decrease in fitness in *Pseudomonas aeruginosa* mutation accumulation lines was explained by only 4.5% of the mutational steps that had a highly deleterious effect on fitness, only 0.5% of all mutations fixed. Previous work with an earlier generation of these mutant lines (and the mutant lines of several other *A. thaliana* ecotypes) suggested that beneficial mutations occurred, but that the magnitude of deleterious mutations was greater (Stearns & Fenster, 2016). This study, when considered in the context of the previous *A. thaliana* mutation accumulation studies, suggests that high magnitude deleterious mutations are more common than high magnitude beneficial mutations and that adaptation likely occurs due to small or intermediate effect beneficial mutations, as suggested by Fisher (1930) and Kimura (1983) and supported experimentally (Barrett, MacLean, & Bell, 2006; Heilbron et al., 2014; Sousa, Magalhaes, & Gordo, 2012). The large deleterious mutations that may be affecting these lines would likely be removed from the population via selection and would not contribute significantly to standing genetic variation.

While the spectrum of mutations due to EMS is similar to that from spontaneous mutation (Greene et al., 2003; Ossowski et al., 2010), EMS does not result in indels and is therefore only a subset of natural spontaneous mutations. It is possible that mutations resulting in indels may have a different distribution of effects on fitness than the point mutations induced by EMS. It is difficult to conclude that indels are more likely to be beneficial. Thus, we favor the hypothesis that we observed generally greater deleterious cumulative effects of mutations because we generated many more mutations and deleterious mutations are more likely to have large negative effects on fitness than beneficial mutations having large positive effects on fitness. This may be important to the process of adaptation. If deleterious mutations are more often strongly deleterious, then they are likely to be lost quickly due to selection. The smaller magnitude beneficial mutations, while not as common, are therefore more likely to contribute to adaptation standing genetic variation.

In comparing fitness from field assays (mass (=reproduction) × survivorship) to that under growth room conditions (silique number), we see that there is a significant line effect for both. However, the rank of the founder differs in both. Under field conditions, all but one line performed worse than the founder (Figure 2). Under artificial grow room conditions, six of the 20 mutant lines outperformed the founder (Figure 4). We believe this reflects the fact that there are more perturbations under field conditions and that more of the induced mutations are affecting fitness. Under the relatively benign artificial conditions, fewer mutations affect fitness (in fact survivorship was not even a factor) and variation due to mutations in the fitness component (siliques) presented were similar to the quantitative traits that are not as closely tied to fitness. Kondrashov and Houle (1994) also observed greater mutation accumulation line effects in fly populations exposed to stressful competitive environments consistent with either more mutations expressed or mutations having greater effect. Likewise, the genetic load may be more detrimental under more stressful conditions (Frankham, 2015). This highlights the importance of investigating the fitness effects of new mutations under more natural conditions, as the difference between that and artificial conditions can be nontrivial. It is therefore not surprising that we find a significant number of lines with reduced fitness under field conditions and not under artificial conditions.

### 4.2 Other quantitative traits

Traits that are not as closely related to fitness are expected to be bidirectionally affected by new mutations. Three other traits were investigated under artificial conditions (Julian day of first flower, number of trichomes per midrib length, and side branch/main branch mass). These traits are likely less closely tied to fitness with the exception of branching (Lortie & Aarssen, 2000), particularly under artificial conditions. Branching for an annual plant will be associated with more reproductive meristems and therefore more flowers and fruit. Significant mutant line effects were found for all the traits except side branch/main branch mass. Although mutations produced a significant line effect in two of the traits that were not as related to fitness (day of first flowering, number of trichomes per midrib length), they were no more likely to decrease the trait value than increase it, as expected. This was also true of the trait most closely related to fitness (silique number), despite predictions about the effects of new mutations on fitness components, and contrary to the results from the field study.

While the traits flowering time and the presence or absence of trichomes have been shown to be under major gene control (Johanson et al., 2000; Karkkainen & Agren, 2002 and Marks, 1997; Shindo et al., 2005; respectively), our results corroborate that these traits also have a polygenic component (Samis et al., 2012; Symonds et al., 2005; Wilczek et al., 2009). For example, flowering time has evolved across an east–west gradient in North American invasive *A. thaliana* independent of the alleles at the major flowering time loci (Samis et al., 2012). Trichome density has been tied to at least nine QTL (Symonds et al., 2005). Thus, we conclude that despite some traits evolving through substitutions at major loci, mutation effects on polygenic loci may also contribute to standing genetic variation for these traits, and hence, also to a selection response. Furthermore, the lack of correlation in the effects of mutations on the traits suggests that these traits may be able to evolve independently, although we cannot rule out a failure to detect correlations due to low sample size. This lack of correlation in expression of the traits across the mutant lines also supports the notion that the EMS approach led to mutations throughout the genome.

## 5 Conclusion

The results of this study are congruent with the mainstream view of the effect of mutations on fitness in that most of the lines decreased fitness relative to a premutation founder and that effect was more pronounced under field conditions. This is contrasted to previous recent results from *A. thaliana*, but strays from those results in an explicable way. Increasing the number of mutations by three‐ to fourfold increases the likelihood that a strongly deleterious mutation will occur and counter the effects of slightly beneficial mutations. Fisher used the analogy of focusing a microscope. If a specimen is reasonably near focus, then large movements with the course adjustment are more likely to reduce focus than small changes with the fine focus (Fisher, 1930). While our study may be somewhat misleading with regard to how adaptation actually occurs, due to the high number of mutations induced, our study can inform us about the ability of new mutations to contribute to adaptation. This study confirms the widely held belief that new mutations are able to contribute more to quantitative traits that are not close to fitness than they are to traits more directly related to fitness, particularly under field conditions. Thus, we show that new mutations may contribute to standing genetic variation.

## Conflict of interest

None declared.
